# Supplementary material for: Progress on SDG 7 achieved by EU countries in relation to the target year 2030: A multidimensional indicator analysis using dynamic relative taxonomy
Source: PLoS One. 2024 Feb 28;19(2):e0297856. doi: 10.1371/journal.pone.0297856 (PMC10901328; doi:10.1371/journal.pone.0297856)
Supplement: Attachment — Submitted filename: Reviewer_3_response_04_01_2024.docx [file pone.0297856.s001.docx]

**Answers to the comments of the Reviewer 3 on the paper:**

## “Progress on SDG 7 achieved by EU countries in relation to the target year 2030: a multidimensional indicator analysis using dynamic relative taxonomy”

We would like to thank the Reviewer for valuable comments, which have been taken into account in the revised version of the article.

**Please find below the answers to each comment**

**Comment 1.**

*The introduction contains a lot of general information about SDG 7 and its indicators. To improve the introduction, the authors should focus on clearly stating the objective of the study. It would have been beneficial to add a few sentences that briefly introduce the research problem, objectives, novelty and significance of the study.*

Answer.

The remark has been taken into account in the corrected version of the article. Section 1.2 introduces the following supplemented corrected text in red:

The purpose of the following study is to assess progress made by individual EU countries in 2010-2021 towards achieving SDG 7 and to determine their distance in relation to targets set for 2030. ~~To this end, the authors propose an aggregate index constructed by applying dynamic relative taxonomy (part 3 of the article), which is used to determine the distance of individual EU countries in relation to targets set for 2030.~~ To overcome the problems with the first and second approach, we propose an innovative third approach, which accounts for the target values of the indicators and uses adjusted data. This approach has not been used in this type of research so far and the use of the geometric mean in the dynamic relative taxonomy method has made it possible to reduce the impact of the negative compensation effect on the values of aggregate measures. The results of the study have important implications for individual EU countries. In addition to showing progress towards the SDG 7 target, they also represent the distance that separates each country from achieving the the 2030 target. The proposed methodology can also be used to assess progress made by EU countries in the implementation of other SDGs of the 2030 Agenda.

**Comment 2.**

*The literature review section is well written and provides a comprehensive overview of previous research. However, it is necessary to highlight the research gap that this study addresses. The authors should highlight why their study is important in relation to the existing literature. What specific gaps or limitations of previous studies does this research seek to overcome?*

Answer.

The remark has been taken into account in the corrected version of the article. Gaps and limitations of previous studies on the assessment of SDG7 are presented in Section 1.2. This is reflected in the revised version of the text with regard to note 1. In addition, the last paragraph of Section 2 has been replaced with the following text highlighted in red:

While the importance of assessing progress towards achieving sustainable development goals is recognized, the number of articles proposing new methods, especially those representing a dynamic approach, is still rather limited. In the case of the SDGs, given the large number of indicators, it is reasonable to opt for composite indicators [10]. Composite indicators are associated with different degrees of compensation. One can distinguish fully compensatory, partially compensatory, or non-compensatory indicators (see section 1.2). The recommended approach is to assess sustainability in relation to a threshold or a target [10, 72]. According to [73], ‘a given indicator does not say anything about sustainability, unless a reference value such as thresholds is given to it’. Non-compensatory methods can be used to identify weak points in the global assessment of an object and thus possible areas for improvement. ~~For these reasons, we have chosen to use a multidimensional indicator based on the principles of dynamic relative taxonomy, which can incorporate reference levels for each indicator and be used to rank countries accordingly.~~ For these reasons, we propose a novel non-compensatory approach based on principles of dynamic relative taxonomy applied to the procedure of constructing an aggregate measure. It can account for reference levels of each indicator and be used to rank countries accordingly showing the varying distance that separates individual EU countries from the targets set out in Agenda 2030. An additional advantage of this method is that the dynamic approach indicates not only relations between the objects in specific periods, but also changes in the phenomenon of interest that took place between objects over the entire reference period. Thus, it can be used to track cross-sectional and longitudinal changes.

**Comment 3.**

*It is appropriate to include a composite indicator analysis for the EU as a whole. This analysis should provide insight into how the European Union as a whole is progressing towards SDG 7.*

Answer.

The remark has been taken into account in the corrected version of the article.

In section 4 the following text, highlighted in red, has been added:

The European Union as a whole is making progress towards meeting the SDG 7 goal. The improvement in the value of the aggregate measurer ${SM}_{it}$ in the period 2010-2021 was $\Delta1=-0.1878$. In the Eurostat report [84], progress towards the SDG 7 goal was described as moderately favorable.

In section 4 the following text, highlighted in red, has been added:

The distance of the European Union as a whole in 2021 in relation to the target set for 2030 is $\Delta1=-0.4207$. Fourteen EU countries were closer to the target in 2021 (see Figure 1).

The following item has been added to the references:

84. Eurostat. Sustainable Development in the European Union: monitoring report on progress towards the SDGs in an EU context – 2023 edition. Luxembourg: Eurostat. 2023. <https://ec.europa.eu/eurostat/web/products-flagship-publications/w/ks-04-23-184> (accessed 12 December 2023).

**Comment 4.**

*The authors should compare and discuss the results of their study with the results of other similar studies in the field of research. This will help readers to understand how the findings of this paper match or differ from existing research. Also, a discussion of the differences between countries will offer valuable insights into regional development.*

Answer.

There a number of reasons why it is not possible to directly compare our results with those obtained in other studies (mentioned in the literature review in section 1.2 and section 2):

–	different reference periods and sets of objects used in those studies,

–	the inclusion of a larger or smaller number of variables than those used to measure progress on SDG 7 achieved by EU countries,

–	different approaches used. Section 1.2 describes three approaches. Since the first approach does not account for the target values these indicators should achieve by 2030, it cannot be used to determine the distance of individual EU countries in relation to targets set for 2030. The second approach takes into account the target values of the indicators without data adjustment. The articles cited in section 1.2 present the first and second approaches. The disadvantages of the first approach include the fact that the target values of the indicators for 2030 are not taken into account (it is not possible to determine the distance of individual EU countries in relation to the target values); the data are not corrected – the values of aggregate measures are distorted (owing to the compensation effect). The use of non-corrected data is also the main disadvantage of the second approach. To overcome these problems, we propose an innovative third approach, which accounts for the target values of the indicators and uses adjusted data. The data are corrected as follows: when a given country exceeds the EU-level targets for 2030, these higher national values (in the case of stimulants) or lower national values (in the case of destimulants) are not included in the analysis but are replaced by target values set for the entire European Union. In this way, the drawbacks of the first and second approaches are eliminated.

**Comment 5.**

*In the final section, the authors should highlight the policy implications of their study findings. For example, they could highlight which indicators were most affected by events such as the pandemic and provide recommendations for policy responses or preventive measures. In addition, it is essential to discuss how development policies can be shaped to avoid a return to pre-crisis patterns. This will make the document more practical and valuable for policy makers.*

Answer.

The remark has been taken into account in the corrected version of the article. The fifth paragraph in Section 5 has been replaced by the following text in red:

~~Because of the COVID-19 pandemic, progress on SDG 7 achieved by Sweden, Denmark, and Germany in 2020 was worse than that observed in 2019.~~ The trend regarding the implementation of the 2030 Agenda has been influenced by the COVID-19 pandemic. Many EU countries experienced a slowdown in the progress towards SDGs. In the case of SDG 7, the distance from the 2030 target in 2020 compared to 2019 had increased for Sweden, Denmark, and Germany. A similar deterioration in performance could be observed ~~in 2021 in relation to 2020~~ from 2020 to 2021 in the case of 16 countries (Austria, Finland, Netherlands, France, Czechia, Croatia, Ireland, Romania, Italy, Spain, Poland, Slovakia, Belgium, Hungary, Lithuania, Bulgaria). ~~No negative effects during the pandemic can be observed for the remaining 8 countries.~~ It is worth emphasizing that during the pandemic, some improvement could be observed for certain indicators. Counter-pandemic measures helped to enhance energy efficiency, which is one of the key pillars in achieving SDG 7. The restrictions on public life and lower economic activity reduced energy consumption from 2019 to 2020 by more than 8%. The reduction in final energy consumption also resulted in greater energy supply and increased the share of renewables in gross final energy consumption. The economic recovery of 2021 and the return to pre-pandemic mobility patterns increased the demand for energy again. However, consumption remained below pre-pandemic levels as the effects of the pandemic continued to shape energy and economic activities. The most obvious negative consequence of the pandemic was the increase in energy consumption by EU households. Given these short-term trends, it is clear that in order to ensure the EU achieves its goals by 2030, changes in all indicators should be continuously monitored and assessed. The main motivation of our study was therefore to propose a methodological approach that could provide information to make such monitoring possible.
